# Integrated Multicriteria Decision-Making Methods to Solve Supplier Selection Problem: A Case Study in a Hospital

**DOI:** 10.1155/2019/5614892

**Published:** 2019-10-10

**Authors:** Serap Akcan, Meral Güldeş

**Affiliations:** ^1^Industrial Engineering, Suleyman Demirel University, Isparta 32260, Turkey; ^2^Industrial Engineering, Cumhuriyet University, Sivas 58040, Turkey

## Abstract

In supply chain literature, supplier evaluation and selection problem is one of the most studied subjects because of the significant roles of suppliers in terms of the chain's sustainability and profitability. Therefore, it is important for organizations to adopt a systematic way to evaluate and select the best supplier according to their respective criteria in today's competitive environment. Multicriteria decision-making methods provide for this need of organizations because determination of an appropriate supplier selection is a multicriteria decision-making (MCDM) problem essentially. Although a lot of applications of these methods for supplier evaluation and selection can be seen in the literature, studies in the health-care sector are insufficient. Hospitals in the health-care sector also have to consider their supplier-related decisions to decrease risks and threads which affect their effectiveness. The aim of this study was to fill this gap by providing different hybrid models for selecting the best supplier for hospitals. Supplier evaluation and selection process start with recognizing the related criteria according to the studies in the literature. Analytic hierarchy process (AHP) method is deployed to weight the criteria, and suppliers are listed via technique for order preference by similarity to ideal solution (TOPSIS), elimination and choice translating reality English (ELECTRE), grey relational analysis (GRA), and simple additive weighting (SAW) methods. The main aim of this study was to present different hybrid MCDM methods and show their efficiency and consistency with each other. In this study, hybrid multicriteria decision-making models (AHP-TOPSIS, AHP-ELECTRE, AHP-GRA, and AHP-SAW) are presented and compared. The results show that the presented hybrid methods in this study are consistent with each other and give the same ranking for the selection of the best supplier. It can be considered as a useful guideline for hospitals.

## 1. Introduction

In a rapidly changing, competitive environment, enterprises should cooperate with suppliers meeting their goals in order to respond more rapidly and more accurately to the changing customer needs. In the supply chain management process, the company choosing an appropriate supplier becomes more advantageous in terms of competition compared to the other enterprises. In this respect, supplier selection is an important issue.

When the literature is reviewed, several studies regarding supplier selection could be found. However, these studies are overwhelmingly performed in automotive and electronic sectors [1, 2]. Because of the fact that delays cause patient discontent and high cost, meeting patients' needs on time has great importance in the health-care sector [3, 4]. Therefore, supplier selection is an essential issue in the healthcare sector as well. A review of the literature showed that the number of studies using hybrid multicriteria decision-making (MCDM) methods on supplier selection problem is limited.

Lambert et al. [5] examined supplier selection criteria in the health-care industry. They presented quality, price, delivery, and service as the commonly used criteria for supplier selection in the health-care area. Ho et al. [6] reviewed the literature from 2000 to 2008 using multicriteria decision-making methods for supplier selection. They observed the most frequently used method is data envelopment analysis and the most popular integrated method is analytical hierarchy process-goal programming. They also presented the most popular criterion in supplier selection problem. Khodadadzadeh and Sadjadi [7] reviewed the literature between the years 2000 and 2013 where data envelopment analysis (DEA) applications were used in different industries for supplier selection, and presented that 60% is related to business, whereas 4% is related to healthcare. Chai et al. [8] conducted a systematic literature review between the years 2008 and 2012 on various applications of decision-making methods for supplier selection. They reviewed 123 articles in detail and outlined 26 applied decision-making methods according to three perspectives: multicriteria decision-making, mathematical programming, and artificial intelligence. Moreover, Chen and Wu [9] developed a modified failure mode and effects analysis (FMEA) method to select new suppliers and used the analytic hierarchy process (AHP) method to determine the weight of each criterion for supplier selection. They presented a case study in the semiconductor industry and determined quality, cost, deliverability, service, technology, and productivity as the supplier selection criteria. In addition, Schmidt et al. [10] presented analytical hierarchy process applications in health-care research from 1981 to 2015. Furthermore, Bahadori et al. [11] established a model for selecting the best supplier in a military hospital using a combination of artificial neural network and fuzzy multicriteria optimization and compromise solution (VIKOR). They stated that “quality” was the most important criterion for supplier selection in their study. Kim et al. [12] presented a mathematical model and a branch-and-bound algorithm to buyers' planning problem. They tested the accuracy of the proposed algorithm by performing computer experiments and obtained a near-optimal solution. Mari et al. [13] proposed a fuzzy-based multiobjective approach and optimization solution methodology to develop resilient criteria for supplier selection. Stević et al. [14] presented a study for supplier selection in which they used the decision-making trail and evaluation laboratory (DEMATEL) method to obtain weight coefficients and the rough evaluation based on distance from average solution (EDAS) method. Wang et al. [15] proposed a hybrid MCDM model for supplier selection problem. Fuzzy AHP and green DEA methods are used to develop the model. The weights of criteria are computed by these methods and potential suppliers are ranked by using green DEA.

The main contribution of this study is that it provides a useful guideline for supplier evaluation and selection problem in the health-care sector by proposing the hybrid MCDM methods and fills this gap. It is possible to see a few hybrid models used in different studies for supplier selection, but this study presents several methods and gives a comparative analysis of the generated methods. It also shows consistency among the proposed methods and encourages decision makers to deploy the most suitable method for their limitations.

The study consists of four sections. The literature reviews are given in the Introduction section. In the Methods section, the proposed method is examined and methods will be discussed. Then, a case study is presented to illustrate the proposed method. Finally, conclusions are stated.

## 2. Methods

**Figure 1:**
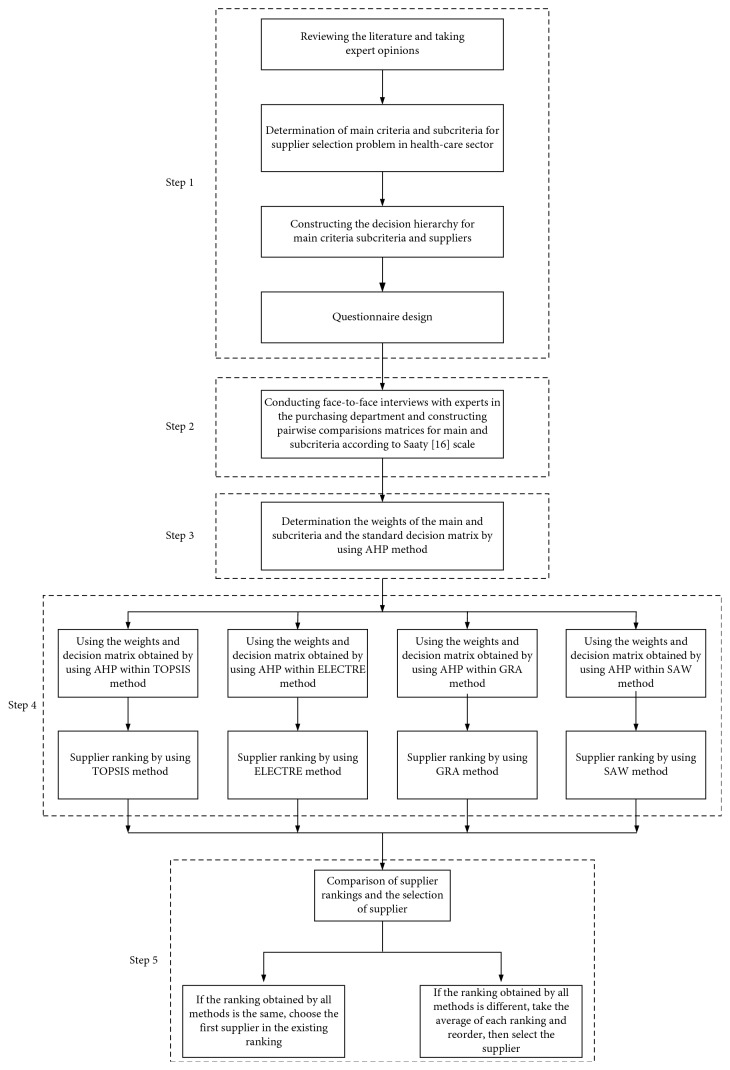
Framework of the proposed method.

This study aims to present integrated multicriteria decision-making methods consisting of analytic hierarchy process (AHP), technique for order preference by similarity to ideal solution (TOPSIS), elimination and choice translating reality English (ELECTRE), grey relational analysis (GRA), and simple additive weighting (SAW) for determining the best supplier in the healthcare sector and overcoming the gap in the literature. This study consists of five steps given in Figure 1. In the first step, the literature was reviewed in order to determine the main criteria and subcriteria for evaluating and selecting the best supplier in the healthcare sector. Then, the decision hierarchy was constructed and questionnaire was designed. In the second step, face-to-face interviews were performed with purchasing experts to evaluate the suppliers. In the third step, the weights of criteria and the decision matrix were found using the AHP method. In the fourth step, hybrid MCDM models (AHP-TOPSIS, AHP-ELECTRE, AHP-GRA, and AHP-SAW) were used to select the best supplier. Finally, in the fifth step, the hybrid models were compared.

The MCDM method is a branch of a general class of Operations Research models. MCDM methods can easily and successfully solve the evaluation and selection problems, which are complicated and have multiple contradictory objectives or criteria. In many real-life decision-making problems, especially for supplier selection, MCDM methods are frequently used. The recent trend is using the MCDM methods integrating two or more methods. In this study, AHP, TOPSIS, ELECTRE, GRA, and SAW were used for supplier selection in a hospital. These methods are explained in the following section.

### 2.1. Analytic Hierarchy Process (AHP)

The AHP proposed by Saaty [16] is one of the frequently used MCDM methods. A hierarchy is identified to show the target on the top and alternatives on the bottom. Then, pairwise comparisons and pairwise comparison matrixes for the criteria in each level are obtained according to Saaty's scale [16]. According to the formula CI=(*λ*_max_ − *n*)/(*n* − 1), (*λ*_max_: largest eigenvalue), the consistency index (CI) is computed. Afterward, by taking random index (RI) values from Table 1, the consistency ratio (CR) using the formula CR= CI/RI is computed for each matrix to measure whether the relative estimation is viable. The values of CR should be less than or equal to 0.10 for each matrix.

### 2.2. Technique for Order Preference by Similarity to Ideal Solution (TOPSIS)

TOPSIS, proposed by Hwang and Yoon [17], is one of the MCDM methods. In this method, the positive ideal is produced of all best values obtainable from the criteria, whereas the negative ideal solution is produced of all worst values obtainable from the criteria [18]. The following steps can be described for using TOPSIS [19–21]:

*Obtaining the decision matrix*:

(1) $$\begin{array}{c} X_{ij}=\left[\begin{array}{ccc} x_{11} & \cdots & x_{1n}\\ \vdots & \vdots & \vdots \\ x_{m1} & \cdots & x_{mn} \end{array}\right]. \end{array}$$

*Obtaining the normalized decision matrix* (for *i*=1,…, *m*, *j*=1,…, *n*):

(2) $$\begin{array}{c} R_{ij}=\left[\begin{array}{ccc} r_{11} & \cdots & r_{1n}\\ \vdots & \vdots & \vdots \\ r_{m1} & \cdots & r_{mn} \end{array}\right], \end{array}$$

(3) $$\begin{array}{c} r_{ij}=\frac{x_{ij}}{\sqrt{\sum_{i=1}^{m}x_{ij}^{2}}}. \end{array}$$

*Obtaining the weighted decision matrix (the sum of the weights should be 1)*:

(4) $$\begin{array}{c} V_{ij}=R_{ij}\;*\;W_{n\times n}=\left[\begin{array}{ccc} v_{11} & \cdots & v_{1n}\\ \vdots & \vdots & \vdots \\ v_{m1} & \cdots & v_{mn} \end{array}\right]. \end{array}$$

*Obtaining the positive ideal (A* ^+^ *) and negative ideal (A* ^−^ *) values*:

(5) $$\begin{array}{c} A^{+}\;=\;\left\{v_{1}^{+},v_{2}^{+},\ldots ,v_{n}^{+}\right\}, \end{array}$$

(6) $$\begin{array}{c} A^{-}\;=\;\left\{v_{1}^{-},v_{2}^{-},\ldots ,v_{n}^{-}\right\}. \end{array}$$

*Obtaining S* ^*+*^ *and S* ^*−*^ *(separation measures)*:

(7) $$\begin{array}{c} S_{i}^{+}\;=\;{\left[\overset{n}{\underset{j=1}{\sum }}{\left(v_{ij}-v_{j}^{+}\right)}^{2}\right]}^{1/2}, \end{array}$$

(8) $$\begin{array}{c} S_{i}^{-}\;=\;{\left[\overset{n}{\underset{j=1}{\sum }}{\left(v_{ij}-v_{j}^{-}\right)}^{2}\right]}^{1/2}. \end{array}$$

*To rank orders, obtaining the relative closeness*:

(9) $$\begin{array}{c} C_{i}^{+}=\frac{S_{i}^{-}}{S_{i}^{+}+S_{i}^{-}}\;,\;0\leq C_{i}^{+}\leq 1. \end{array}$$

### 2.3. Elimination and Choice Translating Reality English (ELECTRE)

ELECTRE is a multicriteria decision-making method suggested by Roy [22, 23]. Different versions of ELECTRE methods (choosing, ranking, and sorting) can be found. ELECTRE I, Iv, and IS are for choosing the decision problem, ELECTRE II, III, and IV is for ranking the decision problem, and ELECTRE TRI is for sorting the decision problem [24]. Steps of ELECTRE are defined below [25].

*Decision matrix (A) is formed*:

(10) $$\begin{array}{c} A_{ij}=\left[\begin{array}{cccc} a_{11} & a_{12} & \cdots & a_{1n}\\ a_{21} & a_{22} & \cdots & a_{2n}\\ \cdot & & & \cdot \\ \cdot & & & \cdot \\ \cdot & & & \cdot \\ a_{m1} & a_{m2} & \cdots & a_{mn} \end{array}\right]. \end{array}$$

In matrix *A*_*ij*_, *m* gives the number of decision points and *n* gives the number of factors.

*The standard decision matrix (X) is formed*:

(11) $$\begin{array}{c} x_{ij}=\frac{a_{ij}}{\sqrt{\sum_{k=1}^{m}a_{kj}^{2}}}. \end{array}$$

For example, to compute the element *x*_11_ of matrix, *a*_11_ element of matrix *A* is divided by the square root of sum squares of elements of column 1. End of the calculations matrix *X* is acquired as follows:

(12) $$\begin{array}{c} X_{ij}=\left[\begin{array}{cccc} x_{11} & x_{12} & \cdots & x_{1n}\\ x_{21} & x_{22} & \cdots & x_{2n}\\ \cdot & & & \cdot \\ \cdot & & & \cdot \\ \cdot & & & \cdot \\ x_{m1} & x_{m2} & \cdots & x_{mn} \end{array}\right]. \end{array}$$

*Weighted standard decision matrix (Y) is formed*:

First of all, decision makers should determine the weights (*w*_*i*_) of the evaluation criteria (∑_*i*=1_^*n*^*w*_*i*_=1).

(13) $$\begin{array}{c} Y_{ij}=\left[\begin{array}{cccc} w_{1}x_{11} & w_{2}x_{12} & \cdots & w_{n}x_{1n}\\ w_{1}x_{21} & w_{2}x_{22} & \cdots & w_{n}x_{2n}\\ \cdot & & & \cdot \\ \cdot & & & \cdot \\ \cdot & & & \cdot \\ w_{1}x_{m1} & w_{2}x_{m2} & \cdots & w_{n}x_{mn} \end{array}\right]. \end{array}$$

*Determination of the concordance (C*_*kl*_ *) and discordance (D*_*kl*_ *) sets*:

For determining the concordance sets, matrix *Y* is used, decision points are compared to each other in terms of evaluation criteria, and matrices are defined with the formula shown below:

(14) $$\begin{array}{c} C_{kl}=\left\{j,y_{kj}\geq y_{lj}\right\}. \end{array}$$

Basically, the formula depends on comparison of sizes of row elements. In a multiple decision problem, the number of concordance sets is (*m* · *m* − *m*); to constitute the concordance set, *k* and *l* indices should be *k* ≠ *l*. Number of elements in a concordance set can be equal to the number of evaluation criteria (*n*) at maximum.

*Concordance (C) and discordance matrices (D) are formed*:

For constituting the concordance Matrix (*C*), concordance sets are used. Matrix *C* has *m* × *m* dimensions and does not take any value for *k*=*l*. Elements of the matrix *C* are computed by the relation in the formula shown below.

(15) $$\begin{array}{c} C_{kl}=\underset{j\in C_{kl}}{\sum }w_{j}. \end{array}$$

Matrix *C* is shown below:

(16) $$\begin{array}{c} C=\left[\begin{array}{ccccc} - & c_{12} & c_{13} & \cdots & c_{1m}\\ c_{21} & - & c_{23} & \cdots & c_{2m}\\ \cdot & & & & \cdot \\ \cdot & & & & \cdot \\ \cdot & & & & \cdot \\ c_{m1} & c_{m2} & c_{m3} & \cdots & - \end{array}\right]. \end{array}$$

Elements of the discordance matrix (*D*) are computed with the formula shown below:

(17) $$\begin{array}{c} d_{kl}=\frac{\max_{j\in Dk\;l}\left|y_{kj}-y_{lj}\right|}{\max_{j}\left|y_{kj}-y_{lj}\right|}. \end{array}$$

Matrix *D* is pointed out below:

(18) $$\begin{array}{c} D=\left[\begin{array}{ccccc} - & d_{12} & d_{13} & \cdots & d_{1m}\\ d_{21} & - & d_{23} & \cdots & d_{2m}\\ \cdot & & & & \cdot \\ \cdot & & & & \cdot \\ \cdot & & & & \cdot \\ d_{m1} & d_{m2} & d_{m3} & \cdots & - \end{array}\right]. \end{array}$$

*Concordance strength (F) and discordance strength (G) matrices are formed*:

The concordance strength matrix (*F*) has *m* × *m* dimensions, and elements of this matrix are acquired from the comparison of concordance threshold value ($\underline{c}$) with the elements of the concordance matrix (*c*_*ki*_). The concordance threshold value $\left(\underline{c}\right)$ is obtained by the formula shown below:

(19) $$\begin{array}{c} \underline{c}=\frac{1}{m\left(m-1\right)}\overset{m}{\underset{k=1}{\sum }}\overset{m}{\underset{l=1}{\sum }}c_{kl}, \end{array}$$

*m* in the formula shows the number of decision points. More precisely, $\underline{c}$ value is equal to multiplication of 1/[*m*(*m* − 1)] and sum of elements composing matrix *C*. Elements of matrix *F*(*f*_*kl*_) take either 1 or 0 value; and furthermore, there are no values because the same decision points are shown on the diagonal of the matrix. If $c_{kl}\geq \underline{c}$, then *f*_*kl*_=1; if $c_{kl}<\underline{c}$, then *f*_*kl*_=0. The discordance strength matrix (*G*) has *m* × *m* dimensions and it is formed in a similar way to matrix *F*. The discordance threshold value ($\underline{d}$) is acquired with the help of the formula shown below:

(20) $$\begin{array}{c} \underline{d}=\frac{1}{m\left(m-1\right)}\overset{m}{\underset{k=1}{\sum }}\overset{m}{\underset{l=1}{\sum }}d_{kl}. \end{array}$$

In other words, $\underline{d}$ value is equal to multiplication of 1/[*m*(*m* − 1)] and sum of elements that consist matrix *D*. Elements of matrix *G*(*g*_*kl*_), take either 1 or 0 value; and furthermore, there are no value because the same decision points are shown on the diagonal of the matrix. If $d_{kl}\geq \underline{d}$, then *g*_*kl*_=1; if $d_{kl}<\underline{d}$, then *g*_*kl*_=0.

*Total domination matrix (E) is formed*:

Elements of the total domination matrix (*e*_*kl*_) is equal to the reciprocal multiplication of *f*_*kl*_ and *g*_*kl*_ elements, as shown in the following formula. Herein, matrix *E* has *m* × *m* dimensions correlated to matrices *C* and *D*, and it forms either 1 or 0.

*Determining the importance order of decision points*:

Rows and columns of matrix *E* show the decision points. For example, if matrix *E* is computed as below,

(21) $$\begin{array}{c} E=\left[\begin{array}{ccc} - & 0 & 0\\ 1 & - & 0\\ 1 & 1 & - \end{array}\right], \end{array}$$

then it takes *e*_21_=1, *e*_31_=1, and *e*_32_=1 values. This shows the second decision point's absolute strength to the first decision point, third decision point's absolute strength to the first decision point, and also the third decision point's absolute strength to the second decision point. In this situation, if the decision points are indicated by the *A*_*i*_(*i*=1,2,…, *m*) symbol, importance order of the decision points will occur, such *A*_3_, *A*_2_, and *A*_1_.

### 2.4. Grey Relational Analysis (GRA)

The grey relational analysis method was originally developed by Deng [26]. It is mainly used to solve multiple attribute decision-making (MADM) problems. The GRA method has some advantages. The main advantage of the GRA method is that results are based upon original data. Other advantages are simplicity of calculation, being intelligible, and usefulness. The methodology is outlined below [27].

*Organize original data to enable the comparison*.

Due to the possibility of the data of the original sequence being presented in different units, the sequences should be assimilated into the same status to make accurate comparisons. Here, *x*_0*j*_ is the reference sequence and *y*_*ij*_ is the original data of attribute *j* of alternative *i*. *y*_*ij*_ can be translated into the comparability sequence *x*_*ij*_ using equations (22) and (23). If the larger value is better, it should use equation (22), and if the smaller one is better, it should use equation (23).

(22) $$\begin{array}{c} x_{ij}=\frac{y_{ij}-\text{Min}\left\{y_{ij}\right\}}{\text{Max}\left\{y_{ij}\right\}-\text{Min}\left\{y_{ij}\right\}}, \end{array}$$

(23) $$\begin{array}{c} x_{ij}=\frac{\text{Max}\left\{y_{ij}\right\}-y_{ij}}{\text{Max}\left\{y_{ij}\right\}-\text{Min}\left\{y_{ij}\right\}}. \end{array}$$

*Calculate the difference sequence*.

(24) $$\begin{array}{c} \Delta_{ij}=\left|x_{0j}-x_{ij}\right|. \end{array}$$

*Determine the minimum and maximum difference, and then compute the grey relational coefficient*.

(25) $$\begin{array}{c} \Delta_{\min}\;=\text{ Min}\left\{\Delta_{ij}\right\}, \end{array}$$

(26) $$\begin{array}{c} \Delta_{\max}\;=\text{ Max}\left\{\Delta_{ij}\right\}, \end{array}$$

(27) $$\begin{array}{c} \gamma \left(x_{0j},\;x_{ij}\right)\;=\;\frac{\Delta_{\min}+\zeta \Delta_{\max}}{\Delta_{ij}+\zeta \Delta_{\max}}. \end{array}$$

Here, *ζ* is the distinguishing coefficient *ζ* ∈ [0,1].

*Compute the grey relational score*.

(28) $$\begin{array}{c} \Gamma \left(X_{0},X_{i}\right)=\overset{n}{\underset{j=1}{\sum }}w_{j}\gamma \left(x_{0j},\;x_{ij}\right). \end{array}$$

Here, *w*_*j*_ is the weight of attribute *j* and ∑_*j*=1_^*n*^*w*_*j*_=1.

*Sort the grey relational analysis results*.

### 2.5. Simple Additive Weighting (SAW)

SAW is one of the most commonly preferred MCDM methods. It is based on the weighted average evaluation of the attributes. In the SAW method, each one of the attributes is given a certain weight and each alternative is determined with regard to the corresponding attribute. For each alternative, calculation of an evaluation score is performed by multiplying the scaled value. SAW consists of the following steps [20, 28].

*Obtain the decision matrix*.

*Obtain the normalized decision matrix*.

(29) $$\begin{array}{c} r_{ij}=\left\{\begin{array}{c} \frac{x_{ij}}{\max x_{ij}},\;i=1,\;\ldots ,\;m;\;j=1,\ldots ,\;n,\;\\ \frac{\min x_{ij}}{x_{ij}},\;i=1,\;\ldots ,\;m;\;j=1,\;\ldots ,\;n, \end{array}\right. \end{array}$$

where *x*_*ij*_/max *x*_*ij*_ is used for positive criteria and min*x*_*ij*_/*x*_*ij*_ is used for negative criteria. Here, *x*_*ij*_ is the criterion value, max*x*_*ij*_ is the maximum value for each positive criterion, min*x*_*ij*_ is the minimum value for each negative criterion, and *r*_*ij*_ is the normalized value.

*Get the weighted score for each alternative*.

(30) $$\begin{array}{c} A_{j}=\overset{m}{\underset{j=1}{\sum }}w_{j}r_{ij},\;i=1,\;\ldots ,\;m. \end{array}$$

Here, *x*_*ij*_ is the score of alternative  *i* to criteria *j* and *w*_*j*_ is the weight of criteria *j*.

*Rank the obtained scores*.

## 3. Case Study

**Figure 2:**
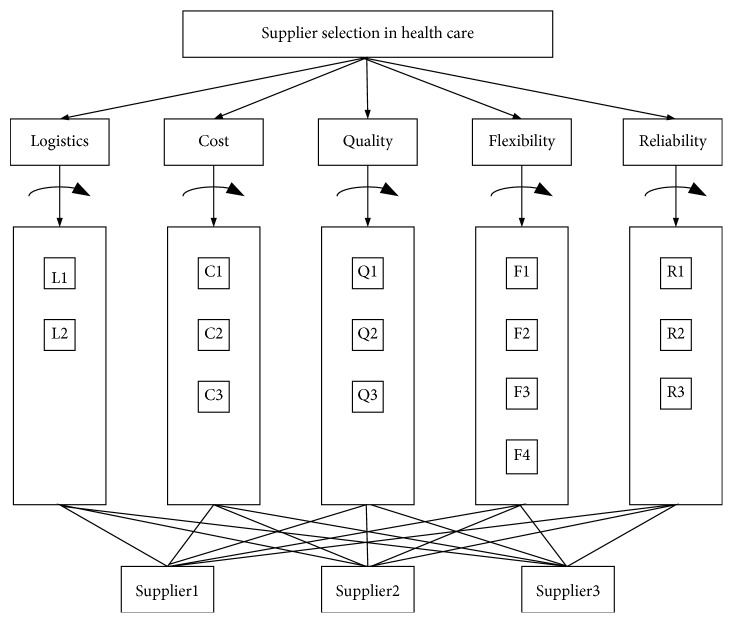
Decision hierarchy for supplier selection.

  Step 1: determining the criteria and construction of the decision hierarchy for supplier selection.
  This study was conducted in a public hospital in Turkey. For selecting the best supplier, the main criteria and subcriteria (Table 2) were determined by considering the literature review. The decision hierarchy for supplier selection is shown in Figure 2. For use in step 2, a questionnaire is designed ([Supplementary-material supplementary-material-1]).
  Step 2: constructing pairwise comparison matrices.
  All of the comparison matrices are given in [Supplementary-material supplementary-material-1].
  Step 3: determination of the weights and standard decision matrix using the AHP method.
  In order to determine the weights of the main criteria and subcriteria, 3 purchasing experts from the examined hospital were interviewed. The experts were asked to evaluate the suppliers (supplier1, supplier2, and supplier3) according to Saaty's 9-point scale. After pairwise comparisons for each subcriterion and each main criterion, the weights of the main criteria and the decision matrix were calculated using AHP (see Table 3). Thus, logistics was found to be the most important criterion, with a priority of 0.513. Criterion cost is also significant with a priority of 0.262. Quality has a priority of 0.129, flexibility has a priority of 0.063, and reliability has a priority of 0.033. The criterion logistics is to meet the expectations of customers at the right time at the right quantity. Therefore, the subcriterion service quality was added under the main criterion logistics. Network organization in logistics, order lead time, and quick response are the other subcriteria for logistics. The criterion cost refers to purchasing prices for each product. The subcriteria for cost are product price, process costs, and quantity rate. The main criterion quality refers to providing the desired quality standards and product specifications. ISO 9000, certifications, and packaging quality are the subcriteria for quality. Flexibility can be described as the easy adaptation of supplier to customer requests and technological developments. The subcriteria are technology, response to changes on demand, to be able to respond to changes in modifications, and to be able to respond to changes in product diversity. Confidence in the supplier affects the working time with the supplier. Therefore, honesty, on-time delivery, and supplying the right product are the subcriteria for the main criterion reliability.

  Step 4: integrated MCDM methods.
  The weights of criteria and decision matrix obtained from the AHP method were integrated into TOPSIS, ELECTRE, GRA, and SAW methods to determine the best supplier for the hospital.

### 3.1. Supplier Selection Using AHP-TOPSIS

The relative closeness and rank orders obtained by using the decision matrix constructed from AHP (Table 3) and the six steps of TOPSIS explained in the Methods section (Equations (1)–(9)) are presented in Table 4. Here, the objective is to maximize the quality, logistics, flexibility, and reliability criteria while minimizing the cost criteria. The sorting results of AHP-TOPSIS are found as Supplier1 > Supplier2 > Supplier3 (Table 4).

### 3.2. Supplier Selection Using AHP-ELECTRE

The total domination matrix *E* which was obtained by using the decision matrix constructed from AHP (Table 3) and the steps of ELECTRE explained in the Methods section (equations (10)–(21)) could be seen below.

(31) $$\begin{array}{c} E=\left[\begin{array}{ccc} - & 0 & 1\\ 0 & - & 0\\ 0 & 0 & - \end{array}\right]. \end{array}$$

As can be seen in the total domination matrix *E*, Supplier2 and Supplier3 have the same priority. On the other hand, Supplier1 has the first priority. Therefore, the sorting results of AHP-ELECTRE are Supplier1 > Supplier2 = Supplier3.

### 3.3. Supplier Selection Using AHP-GRA

Grey relational analysis sorting results obtained by using the decision matrix constructed from AHP (Table 3) and the steps of GRA explained in the Methods section (equations (22)–(28)) are presented in Table 5.

As can be seen in Table 5, it could be noted that Supplier1 has the first highest grey relational score value, Supplier2 has the second highest, and Supplier3 has the third highest grey relational score value. Therefore, the sorting results of AHP-GRA are Supplier1 > Supplier2 > Supplier3.

### 3.4. Supplier Selection Using AHP-SAW

The weighted score for each supplier and ranking orders obtained by using the decision matrix constructed from AHP (Table 3) and the four steps of SAW explained in the case study section (equations (29)–(30)) are shown in Table 6.

As can be seen in Table 6, Supplier1 has the highest weighted score; Supplier2 has the second and Supplier3 has the third highest weighted score. Therefore, the sorting results of AHP-SAW are Supplier1 > Supplier2 >Supplier3.

  Step 5: supplier selection using hybrid MCDM models
  In this study, the weights of criteria and subcriteria were calculated using the AHP method and then, selected MCDM methods were used to select the most suitable supplier. The results for the supplier selection from the generated hybrid methods are shown in Table 7. We can see the consistency among the methods on account of selecting the best supplier. According to the results, researchers can use one of these methods to make a decision. Deployment of the selected method is considered by the researcher and can depend on his/her experience. Easiness of application is also a factor for choosing the method.

As can be seen in Table 7, the hospital should prefer studying with supplier1.

## 4. Conclusions

In today's highly competitive environment, market globalization for each sector increases gradually. As a result, the number of possible suppliers and the number of criteria to deal with when choosing the appropriate supplier also increases. Organizations that can be active either in a manufacturing sector or in a service sector have been imposed to form durable and valid partnerships and select the best supplier for their sustainability. Evaluation and selection of a supplier provides an organization with convenient quality products and/or services at a convenient price, with convenient quantities, and at a convenient time. This process can be highly intricate because it combines a great variety of changeable factors, which can be diversified according to the nature of the products and services to be acquired. It is a crucial function performed by the purchasing department of an organization in order to improve quality and flexibility besides lead time. Because of its importance in terms of an organization, there should be a systematic way for the selection of an appropriate supplier. This process includes the requirement for a new supplier, ascertainment and formulation of the decision criteria, prequalification, final supplier selection, and monitoring of the suppliers selected [29–33].

In the health-care sector, patient care is considered as the most vital objective. However, money is still a key factor for organizations in this sector to achieve their objectives and survive. The cost of delivering healthcare to the public has increased tremendously in recent years. Due to the expanding competition in healthcare, choosing the best supplier among diversified options has become crucial for the sake of attaining customer contentment. Although there is an increase in number of suppliers, the number of stated problems related to lack of quality has also increased seriously. Selecting an efficient supplier allows considerable capability for developing quality while lessening expenses. Because of this fact, many hospitals recognize the importance of selecting their suppliers via a healthy method. Deployment of MCDM methods is an effective way for evaluating and selecting a supplier. This process results in a vast set of criteria and subcriteria to take into account, and lead to a lot of different relations among these criteria and subcriteria, which should be evaluated carefully to make a decision. These methods help the decision makers and provide a more clear comprehension of the problem. These tools consider the viewpoints of several decision makers and assess every decision maker's judgment. Therefore, different tools have been introduced to cope with this problem effectively over a period of time. Investigation of the usage of a specific tool among others requires serious amount of time. Hybridization of different MCDM techniques is a way to address to this problem. In recent years, there has been an increase in the deployment of HMCDM methods to help decision makers. One of the essential reasons for this increment is the acceptance of results attained via these hybrid methods. These methods are also more convenient to deploy for bigger and more intricate problems [34, 35].

In this study, an endeavor has been made to develop some HMCDM methods by mixing AHP, TOPSIS, ELECTRE, GRA, and SAW methods. The methods in this work have addressed the performance evaluation of three different suppliers in the health-care sector and chosen the most suitable supplier. In order to achieve this, several criteria and subcriteria need to be selected from the literature for use in these models. These criteria include logistics, quality, cost, flexibility, reliability, and their related subcriteria. This work does not favor any HMCDM method to other methods, but it emphasizes on the importance of using different techniques to make a decision on the concerned issue. It is also important to check the consistency of the results obtained with various methods [36]. Selection of a specific HMCDM method affects the quality of the decision and the level of endeavor deployed. Results show that Supplier1 is the best option for this problem.

## Figures and Tables

**Table 1:** Random index values [16].

*N*	1	2	3	4	5	6	7	8	9
RI	0	0	0.58	0.9	1.12	1.24	1.32	1.41	1.45

**Table 2:** The main and subcriteria for supplier selection.

Main criteria	Subcriteria
Logistics	(L1) Network organization and order lead time
	(L2) Quick response and service quality

Quality	(Q1) ISO 9000
	(Q2) Certifications
	(Q3) Packaging quality

Cost	(C1) Product price
	(C2) Process costs
	(C3) Quantity discount rate

Flexibility	(F1) Technology
	(F2) Response to changes
	(F3) To be able to respond to changes in modifications
	(F4) To be able to respond to changes in product diversity

Reliability	(R1) Honesty
	(R2) On-time delivery
	(R3) Right product

**Table 3:** Decision matrix.

	Logistics	Quality	Cost	Flexibility	Reliability
Weight	0.513	0.129	0.262	0.063	0.033
Supplier1	0.731	0.292	0.193	0.640	0.086
Supplier2	0.188	0.079	0.203	0.183	0.314
Supplier3	0.081	0.629	0.605	0.177	0.600

**Table 4:** Relative closeness and sorting results.

	*S*_*i*_^+^	*S*_*i*_^−^	Total	*C*_*i*_^+^	Rank orders of AHP-TOPSIS
Supplier1	0.067	0.471	0.539	0.876	1
Supplier2	0.383	0.174	0.557	0.312	2
Supplier3	0.469	0.105	0.574	0.182	3

**Table 5:** Grey relational analysis sorting results.

Weight	0.513	0.129	0.262	0.063	0.033	

Objective	MAX	MAX	MIN	MAX	MAX	
	Logistics	Quality	Cost	Flexibility	Reliability	Grey relational score
Supplier1	0.513	0.058	0.262	0.063	0.011	0.907
Supplier2	0.192	0.043	0.249	0.021	0.016	0.521
Supplier3	0.171	0.129	0.087	0.021	0.033	0.442

**Table 6:** Weighted score for each supplier and ranking orders for AHP-SAW.

	Weighted score	Rank orders of AHP-SAW
Supplier1	0.902	1
Supplier2	0.432	2
Supplier1	0.320	3

**Table 7:** Comparison of the hybrid MCDM methods for supplier selection.

	AHP-TOPSIS	Rank orders	AHP-ELECTRE	Rank orders	AHP-GRA	Rank orders	AHP-SAW	Rank orders
Supplier1	0.876	**1**	1	**1**	0.907	**1**	0.902	**1**
Supplier2	0.312	**2**	0	**2**	0.521	**2**	0.432	**2**
Supplier3	0.182	**3**	0	**2**	0.442	**3**	0.320	**3**

## Data Availability

The data used to support the findings of this study are available from the corresponding author upon request.

## Supplementary Materials

Supplementary Materials For the purpose of constructing pairwise comparison matrices, a questionnaire is designed and added as Appendix 1. All of the comparison matrices are added as Appendix 2.
