# Supplementary material for: Integrated Multicriteria Decision-Making Methods to Solve Supplier Selection Problem: A Case Study in a Hospital
Source: J Healthc Eng. 2019 Oct 10;2019:5614892. doi: 10.1155/2019/5614892 (PMC6811789; doi:10.1155/2019/5614892)
Supplement: Supplementary Materials — For the purpose of constructing pairwise comparison matrices, a questionnaire is designed and added as Appendix 1. All of the comparison matrices are added as Appendix 2. [file 5614892.f1.pdf]

Appendix-1

The main and sub-criteria for supplier selection	Suppliers and their evaluations		
	**Supplier1**	**Supplier2**	**Supplier3**
**Logistics**			
(L1) Network organization and order lead time			
(L2) Quick response and service quality			
**Quality**			
(Q1) ISO 9000			
(Q2) Certifications			
(Q3) Packaging quality			
**Cost**			
(C1) Product price			
(C2) Process costs			
(C3) Quantity discount rate			
**Flexibility**			
(F1) Technology			
(F2) Response to changes			
(F3) To be able to respond to changes in modifications			
(F4) To be able to respond to changes in product diversity			
**Reliability**			
(R1) Honesty			
(R2) On-time Delivery			
(R3) Right product			

Appendix-2

Table 3. Pairwise comparison matrix of the main criteria

	Logistics	Quality	Cost	Flexibility	Reliability
Logistics (L)	1	5	3	7	9
Quality (Q)	1/5	1	1/3	3	5
Cost (C)	1/3	3	1	5	7
Flexibility (F)	1/7	1/3	1/5	1	3
Reliability (R)	1/9	1/5	1/7	1/3	1

Table 4. Pairwise comparison matrix for the sub-criteria with respect to logistics

	L1	L2
L1	1	7
L2	1/7	1

Table 5. Pairwise comparison matrix for the sub-criteria with respect to quality

	Q1	Q2	Q3
Q1	1	1/3	1/5
Q2	3	1	1/3
Q3	5	3	1

Table 6. Pairwise comparison matrix for the sub-criteria with respect to cost

	C1	C2	C3
C1	1	1/5	1/7
C2	5	1	1/5
C3	7	5	1

Table 7. Pairwise comparison matrix for the sub-criteria with respect to flexibility

	F1	F2	F3	F4
F1	1	7	7	3
F2	1/7	1	1	1/5
F3	1/7	1	1	1/5
F4	1/3	5	5	1

Table 8. Pairwise comparison matrix for the sub-criteria with respect to reliability

	R1	R2	R3
R1	1	1/5	1/9
R2	5	1	1/3
R3	9	3	1

Table 9. Pairwise comparison matrix for the sub-criteria with respect to L1

	Supplier 1	Supplier 2	Supplier 3
Supplier 1 (S1)	1	5	7
Supplier 2 (S2)	1/5	1	3
Supplier 3 (S3)	1/7	1/3	1

Table 10. Pairwise comparison matrix for the sub-criteria with respect to L2

	S1	S2	S3
S1	1	5	7
S2	1/5	1	3
S3	1/7	1/3	1

Table 11. Importance weights for logistics

	L1	L2	
Weight	0,875	0,125	
Supplier			Importance Weights
S1	0,731	0,730	0,731
S2	0,188	0,188	0,188
S3	0,081	0,080	0,081

Table 12. Pairwise comparison matrix for the sub-criteria with respect to Q1

	S1	S2	S3
S1	1	9	1
S2	1/9	1	1/9
S3	1	9	1

Table 13. Pairwise comparison matrix for the sub-criteria with respect to Q2

	S1	S2	S3
S1	1	3	1
S2	1/3	1	1/3
S3	1	3	1

Table 14. Pairwise comparison matrix for the sub-criteria with respect to Q3

	S1	S2	S3
S1	1	5	1/5
S2	1/5	1	1/9
S3	5	9	1

Table 15. Importance weights for quality

	Q1	Q2	Q3	
Weight	0,104	0,258	0,636	
Supplier				Importance Weights
S1	0,473	0,428	0,206	0,292
S2	0,052	0,142	0,058	0,079
S3	0,473	0,428	0,735	0,629

Table 16. Pairwise comparison matrix for the sub-criteria with respect to C1

	S1	S2	S3
S1	1	1/3	1/7
S2	3	1	1/3
S3	7	3	1

Table 17. Pairwise comparison matrix for the sub-criteria with respect to C2

	S1	S2	S3
S1	1	1	1/3
S2	1	1	1/3
S3	3	3	1

Table 18. Pairwise comparison matrix for the sub-criteria with respect to C3

	S1	S2	S3
S1	1	1	1/3
S2	1	1	1/3
S3	3	3	1

Table 19. Importance Weights for Cost

	C1	C2	C3	
Weight	0,066	0,218	0,714	
Supplier				Importance Weights
S1	0,087	0,200	0,200	0,193
S2	0,242	0,200	0,200	0,203
S3	0,669	0,600	0,600	0,605

Table 20. Pairwise comparison matrix for the sub-criteria with respect to F1

	S1	S2	S3
S1	1	9	7
S2	1/9	1	1/3
S3	1/7	3	1

Table 21. Pairwise comparison matrix for the sub-criteria with respect to F2

	S1	S2	S3
S1	1	1	1/3
S2	1	1	1/3
S3	3	3	1

Table 22 . Pairwise comparison matrix for the sub-criteria with respect to F3

	S1	S2	S3
S1	1	5	5
S2	1/5	1	1
S3	1/5	1	1

Table 23 . Pairwise comparison matrix for the sub-criteria with respect to F4

	S1	S2	S3
S1	1	1	3
S2	1	1	3
S3	1/3	1/3	1

Table 24. Importance Weights for Flexibility

	F1	F2	F3	F4	
Weights	0,582	0,067	0,067	0,282	
Supplier					Importance Weights
S1	0,785	0,200	0,714	0,428	0,640
S2	0,061	0,200	0,142	0,428	0,183
S3	0,148	0,600	0,142	0,142	0,177

Table 25. Pairwise comparison matrix for the sub-criteria with respect to R1

	S1	S2	S3
S1	1	1/3	1/3
S2	3	1	1
S3	3	1	1

Table 26. Pairwise comparison matrix for the sub-criteria with respect to R2

	S1	S2	S3
S1	1	1/7	1/7
S2	7	1	1
S3	7	1	1

Table 27. Pairwise comparison matrix for the sub-criteria with respect to R3

	S1	S2	S3
S1	1	1/3	1/7
S2	3	1	1/3
S3	7	3	1

Table 28. Importance Weights for Reliability

	R1	R2	R3	
Weight	0,062	0,265	0,671	
Supplier				Importance Weights
S1	0,142	0,066	0,087	0,086
S2	0,428	0,466	0,242	0,314
S3	0,428	0,466	0,669	0,600
